# In Situ Spatial Reconstruction of Distinct Normal and Pathological Cell Populations Within the Human Adrenal Gland

**DOI:** 10.1210/jendso/bvad131

**Published:** 2023-10-25

**Authors:** Rui Fu, Kathryn Walters, Michael L Kaufman, Katrina Koc, Amber Baldwin, Michael R Clay, Kaitlin J Basham, Katja Kiseljak-Vassiliades, Lauren Fishbein, Neelanjan Mukherjee

**Affiliations:** RNA Biosciences Initiative and Department of Biochemistry and Molecular Genetics, University of Colorado School of Medicine at Colorado Anschutz Medical Campus Aurora, Aurora, CO 80045, USA; Computational Biology, New York Genome Center, New York, NY 10013, USA; RNA Biosciences Initiative and Department of Biochemistry and Molecular Genetics, University of Colorado School of Medicine at Colorado Anschutz Medical Campus Aurora, Aurora, CO 80045, USA; RNA Biosciences Initiative and Department of Biochemistry and Molecular Genetics, University of Colorado School of Medicine at Colorado Anschutz Medical Campus Aurora, Aurora, CO 80045, USA; Division of Endocrinology, Metabolism and Diabetes, Department of Medicine, University of Colorado School of Medicine at Colorado Anschutz Medical Campus Aurora, Aurora, CO 80045, USA; RNA Biosciences Initiative and Department of Biochemistry and Molecular Genetics, University of Colorado School of Medicine at Colorado Anschutz Medical Campus Aurora, Aurora, CO 80045, USA; Department of Pathology, University of Colorado School of Medicine at Colorado Anschutz Medical Campus Aurora, Aurora, CO 80045, USA; Department of Oncological Sciences, Huntsman Cancer Institute, University of Utah, Salt Lake City, UT 84112, USA; Division of Endocrinology, Metabolism and Diabetes, Department of Medicine, University of Colorado School of Medicine at Colorado Anschutz Medical Campus Aurora, Aurora, CO 80045, USA; Research Service Veterans Affairs Medical Center, Aurora, CO 80045, USA; Division of Endocrinology, Metabolism and Diabetes, Department of Medicine, University of Colorado School of Medicine at Colorado Anschutz Medical Campus Aurora, Aurora, CO 80045, USA; RNA Biosciences Initiative and Department of Biochemistry and Molecular Genetics, University of Colorado School of Medicine at Colorado Anschutz Medical Campus Aurora, Aurora, CO 80045, USA

**Keywords:** adrenal cortex, primary aldosteronism, spatial transcriptomics, RNA, steroid hormones

## Abstract

The human adrenal gland consists of concentrically organized, functionally distinct regions responsible for hormone production. Dysregulation of adrenocortical cell differentiation alters the proportion and organization of the functional zones of the adrenal cortex leading to disease. Current models of adrenocortical cell differentiation are based on mouse studies, but there are known organizational and functional differences between human and mouse adrenal glands. This study aimed to investigate the centripetal differentiation model in the human adrenal cortex and characterize aldosterone-producing micronodules (APMs) to better understand adrenal diseases such as primary aldosteronism. We applied spatially resolved in situ transcriptomics to human adrenal tissue sections from 2 individuals and identified distinct cell populations and their positional relationships. The results supported the centripetal differentiation model in humans, with cells progressing from the outer capsule to the zona glomerulosa, zona fasciculata, and zona reticularis. Additionally, we characterized 2 APMs in a 72-year-old woman. Comparison with earlier APM transcriptomes indicated a subset of core genes, but also heterogeneity between APMs. The findings contribute to our understanding of normal and pathological cellular differentiation in the human adrenal cortex.

The human adrenal gland consists of an outer cortex responsible for steroid hormone biosynthesis and an inner medulla responsible for catecholamine synthesis. The cortex is concentrically arranged into histologically and functionally distinct regions including the outer capsule, zona glomerulosa (zG), zona fasciculata (zF), and zona reticularis (zR) [1]. The ability of cortical cells to self-renew and differentiate is crucial to normal adrenocortical homeostasis [2]. Dysregulation of molecular pathways controlling cortical cell differentiation dynamics and/or hormonal secretion leads to human diseases including primary aldosteronism (PA) and adrenocortical carcinoma, among others. The current centripetal differentiation model posits that adult stem and progenitor cells in the capsule/subcapsular region differentiate into zG cells that further differentiate into zF cells and then zR cells [3-5]. This model is based on experiments from mouse adrenal glands, which differ in at least 2 cell populations and functions from human adrenal glands [4]. Nevertheless, these and other recent studies have found that the organization of layers and cell populations in the adrenal gland are more dynamic and heterogeneous than previously known.

An important example of cellular heterogeneity within histologic zones of the human adrenal cortex is the discovery of aldosterone-producing micronodules (APMs), formerly called aldosterone-producing cell clusters [6]. APMs are defined as CYP11B2-positive cell clusters that are not discernible from surrounding cells of the capsule and zG by hematoxylin-eosin (H&E) staining [7]. APMs are associated with autonomous aldosterone production, and a subset of APMs may be precursors to aldosterone-producing adenomas (APAs) [8]. Although mouse models of APA exist, none of the models to date produce APMs, suggesting potential alternative pathways and highlighting the need to work with human tissue samples [9]. Interestingly, APMs can be found in normal human adrenal tissue samples and they increase in frequency with age [10, 11]. Understanding the molecular etiology of APMs, as well as APAs, is critical in understanding PA, an underdiagnosed treatable secondary cause of hypertension. In fact, there has been a recent conceptual shift toward recognizing PA as a continuum of autonomous aldosterone production that exists with varying severity even in normotensive individuals and all the way to severely hypertensive individuals [12, 13]. Unrecognized PA leads to cardiovascular disease, myocardial infarction, and stroke.

The overarching goal of this study was to identify supporting evidence for the centripetal differentiation model in the human adrenal cortex and identify pathways involved in APM development to better understand the etiology of PA. To advance our limited understanding of human adrenocortical cell differentiation and heterogeneity, we need to understand how cell populations within the human adrenal gland self-renew and differentiate. Preserving the spatial relationship between cells and using a global unbiased approach is a critical initial step. In this report, we apply spatially resolved in situ transcriptomics to human adrenal tissue sections to better understand the pathways controlling adrenocortical differentiation. To further investigate cellular heterogeneity, using APMs as an example, we characterized the transcriptomes of APMs compared with neighboring zG cells to identify markers able to discriminate between the two. Together, these data provide foundational knowledge to enhance our understanding of both normal and pathological cellular differentiation in the human adrenal cortex.

## Materials and Methods

### Adrenal Gland Processing

With institutional review board approval (COMIRB 15-0516), we worked with the Donor Alliance to obtain normal healthy adrenal tissue attached to donor kidneys that would otherwise be discarded. Donors had already agreed to be organ donors and agreed to share donor tissue for research purposes. Normal adrenal glands were dissected and placed into histidine tryptophan ketoglutarate (HTK) solution. Within 2 hours of receiving the tissue, the gland was cut into sections for fresh-frozen tissue and to create optical coherence tomography (OCT) blocks for storage. Sections of OCT blocks 10 µm thick were cut and used for H&E to ensure a well-preserved tissue to use in further experiments. Adjacent sections were cut and used for spatial transcriptomics and immunohistochemical analysis.

### Spatial Transcriptomics Sample Processing

Frozen samples were OCT-embedded and sectioned at 10 μm on a Cryostar NX70 cryostat (Thermo Fisher Scientific). Capture sections were fixed with methanol, stained with H&E, and imaged on an Evos M7000 (ThermoFisher) with brightfield settings. Capture sections were then permeabilized and processed to generate RNA libraries following the 10× Visium protocol. Libraries were sequenced to a depth of 60 000 read pairs per spot calculated from the image, on a NovasSeq6000 (Illumina) sequencer with 151 × 151 bp runs.

### Spatial Transcriptomics Data Analysis

Sequencing data were processed with Space Ranger (10× genomics, v1.2.1), followed by further analysis using the Seurat (v4.0.1) tool suite in R [14]. Spots were lightly filtered to ensure the number of genes detected fall between 50 and 15 000, and less than 50% of unique molecular identifiers mapped to mitochondrial genes. This resulted in 1050 of 1073, 736 of 739, 1486 of 1488, and 938 of 938 spots being retained in each section, respectively. After initial SCTransform normalization on each sample and principal component analysis on merged data, integration was performed with Harmony [15] (v1.0) using 30 principal components and θ = 2. Uniform manifold approximation and projection (UMAP) dimension reduction and shared nearest neighbor clustering were carried out on 30 principal components, and clustering results at different resolution settings were explored through Clustree [16] (v0.4.3) and 10× Cloupe browser visualizations. Specific cells overlaying H&E regions of interest were manually traced in 10× Cloupe browser and then exported to retrieve barcodes.

Differential ST cluster gene expression was defined by Wilcoxon test as implemented in Presto (https://github.com/immunogenomics/presto), with thresholds of adjusted *P* value less than or equal to .001 and log2 fold change greater than or equal to 0.5. Cell type identity of clusters was defined in 3 ways: (i) manual inspection of key markers, (ii) expert histological annotation by a pathologist of H&E-stained slides, and (iii) Jaccard index calculation of ST marker gene overlap with previously reported single-cell RNA sequencing (RNA-seq) markers from Fu et al using Clustifyr [17]. For both the medulla and zR/medulla, we initially identified 2 subpopulations. However, we collapsed each of the subpopulations to a single population because of the very minimal number of differentially expressed genes between the original 2 subpopulations. Per-cell gene set expression scoring was calculated through a faster R/rust reimplementation of the Seurat::AddModuleScore algorithm (https://github.com/raysinensis/SCoreRust). Pathways were defined by C2: curated gene sets in the Human MSigDB Collections release 7.5.1 [18]. Pseudotime trajectory was inferred with the R package Destiny [19], designating zR cells as the tip of the diffusion branches. *Z* scores for gene expression along pseudotime was calculated as 50 roughly equal cell number bins.

Deconvolution of spatial spots from the Visium platform was carried out using version 1.2.0 of the R package STdeconvolve [20]. The package employs Latent Dirichlet Allocation (LDA) to estimate cell types and their proportions based on mixed gene expression profiles within each tissue spot. These estimated cell-type signatures are referred to as “topics.” We used the STdeconvolve package as described in the documentation to first use the fitLDA() function and then the optimalModel() and getBetaTheta() functions, using default parameters, to extract the fitted model and topics. For subsequent enrichment analysis of these topics against known WNT targets and immune markers, Fisher exact test was applied. This involved comparing the top-ranking genes within each topic against predetermined sets of marker genes. The WNT targets set included *APCDD1*, *AXIN2*, *LEF1*, *LGR5*, and *NKD1* [21], while the immune markers set consisted of *CD33*, *MPO*, *FUT4*, *ELANE*, *CXCR2*, *CD14*, *S100A9*, and *S100A8* [22]—primarily indicators of neutrophils and the innate immune response. Statistical significance was defined as a *P* value less than .05. Additional immune-related genes from other major cell types were also tested but were not found to be statistically significant. To explore a comprehensive range of cell populations, the number of topics was initially increased for each tissue slice up to a maximum of 50. It was subsequently reduced to identify the minimum number of topics (21, 20, 18, and 15 for slide section A, B, C, and D, respectively) that still captured the relevant cell populations when compared to our test sets.

### Histological Analysis of Adrenal Tissue Samples

Adrenal sections were fixed in 10% neutral buffered formalin for 10 minutes. They were then blocked and permeabilized with CAS-Block + 0.2 TritonX (CAS-T) for 30 minutes. Samples were stained overnight at 4 °C with antimouse CYP11B2 (RRID: AB_2650562) [23] diluted 1:1000 in CAS-T. The primary solution was rinsed with TBS+ 0.1% Tween20 (TBS-T) twice. Antimouse immunoglobulin G Alexa Fluor 488 (RRID:AB_10694704) was diluted 1:1000 in TBS-T and the sample was incubated at room temperature for 1 hour. Samples were rinsed with TBS-T and then mounted using VectaShield Vibrance Antifade Mounting Medium with DAPI (4′,6-diamidino-2-phenylindole). Slides were imaged with a 10× lens on a DeltaVision Elite Deconvolution Microscope and stitched together with DeltaVision software. RNAscope of mouse adrenal tissue was performed as previously described [24].

## Results

### Determining the Spatial Relationships Between Human Adrenal Cell Populations

**Figure 1.**
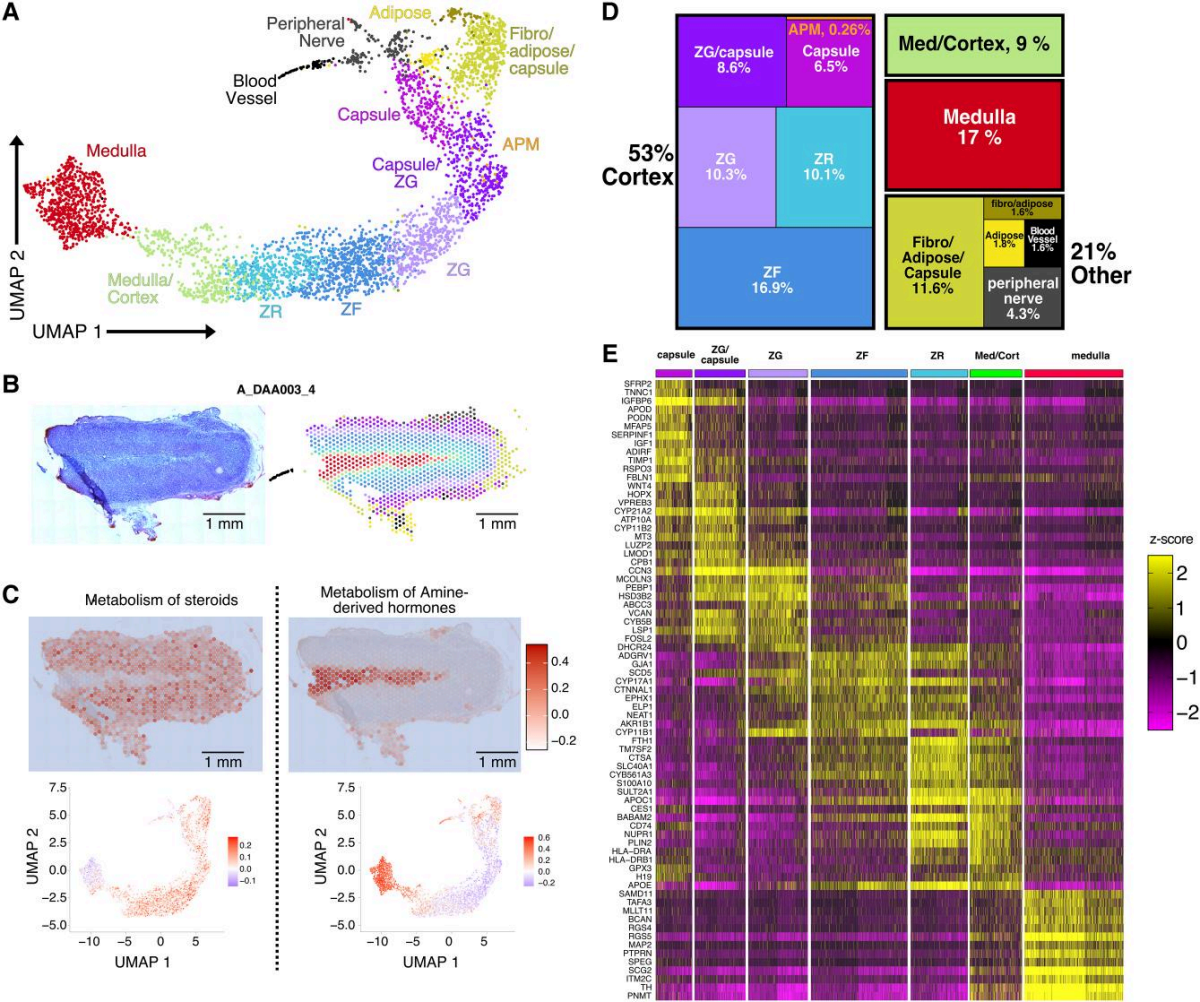
In situ reconstruction of distinct cell populations within the human adrenal gland. A, UMAP projection of harmonized spot data from all 4 adrenal tissue sections color-coded by cell population assignment. B, Hematoxylin-eosin stain (left) of a representative donor adrenal section and in situ spots color-coded by cell population assignment (right). C, Expression of steroid metabolism (left) and amine-derived hormone (right) genes in situ (top) and on the UMAP projection (bottom). D, Tree map of the percentage of spots assigned to specific cell populations. E, Heat map of the *z* scores for the top differentially localized genes.

Visium 10× spatial transcriptome analysis was performed on 4 different normal adrenal sections from 2 individual deceased donors (3 sections from a 31-year-old woman and 1 section from a 72-year-old woman). Harmony was used to integrate the transcriptome data from the 4 sections [23] (Supplementary Fig. S1A and B [25]). On average, approximately 3000 genes per spot were detected for every section (Supplementary Fig. S1C [25]). The UMAP shows the 12 distinct and reproducible cell populations identified based on similarity in gene expression (Fig. 1A and Supplementary Fig. S1D [25]). Projecting the normal adrenal gland cell populations transcriptome back to the H&E-stained tissue section (Fig. 1B, left) revealed the expected concentric organization remarkably consistent with the histology (see Fig. 1B, right). For example, the expression of steroid hormone metabolism genes was highest in the cortical cells determined by visual mapping (Fig. 1C, top left) and assigned cortical zones on UMAP (see Fig. 1C, bottom left). Likewise, the expression of amine-derived hormone gene set was highest in the medullary cells (Fig. 1C, top and bottom right). This observation was true for all 4 tissue sections (see Supplementary Fig. S1D [25]). Next, we assessed the relative contribution of different cell populations to the human adrenal gland based on expression pattern. The adrenal cortex had 53% of the cellular contribution, whereas 17% was medulla, 10% was a mixture of cortex and medulla, and the remaining 21% was composed of fibroadipose tissue, blood vessels, or peripheral nerve cells (Fig. 1D). As expected, genes involved in aldosterone, cortisol, and androgen production were enriched in zG (*CYP11B2*), zF (*CYP11B1*), and zR (*SULT2A1*), respectively (Fig. 1E). We identified known markers of the capsule such as *RSPO3*, as well as known markers of the zG such as *WNT4*. And, *PNMT* and *TH*, which are crucial for catecholamine production, were specifically expressed in the medulla. These results validate our cell population assignments.

### The Centripetal Differentiation Model in the Human Adrenal Cortex

**Figure 2.**
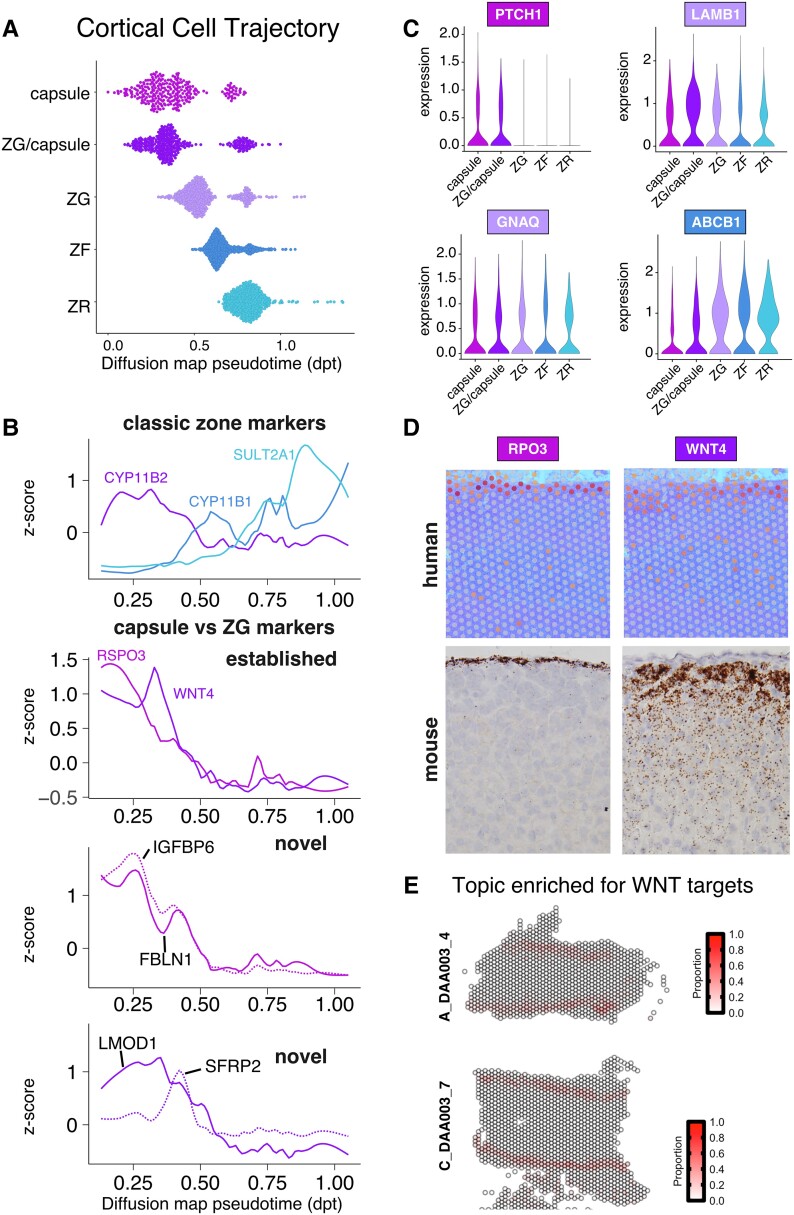
Centripetal differentiation and WNT activity. A, Dot plot of the distribution of diffusion map pseudotime (DPT) values for each adrenocortical cell population. B, Line plot of expression *z* scores vs DPT values for genes colored by their respective cell population. C, Violin plot of the human homologs of the known mouse markers. D, Messenger RNA expression level and pattern of RSPO3 and WNT4 in human adrenal tissue (top). RNAscope analysis of *Rspo3* and *Wnt4* expression in mouse adrenal samples (bottom). E, Heat map of the topic associated with WNT targets.

Next, we examined if the centripetal differentiation model based on studies of the mouse adrenal gland [26, 27] was recapitulated in human adrenal glands. The developmental progression of the identified cortical cell populations was inferred using diffusion pseudotime analysis [28]. This analysis indicated that capsule to zG to zF to zR is the order of differentiation and transition between cell types of the adrenal cortex (Fig. 2A). Classic zone-specific markers for zG (*CYP11B2*), zF (*CYP11B1*), and zR (*SULT2A1*) support the observed order of cell population transitions (Fig. 2B). Furthermore, the peak enrichment of *RSPO3* in the pseudotime regions corresponding to the capsular cells precedes the peak enrichment of *WNT4* in the pseudotime regions corresponding to the zG cells (see Fig. 2B). We also identified novel genes with restricted expression patterns associated with the capsule (*IGFBP6* and *FBNL1*) and capsule/zG (*LMOD1* and *SFRP2*), respectively (see Fig. 2B). The pseudotime values for individual genes were consistent with their expression in cortical cell populations (Supplementary Fig. S2A [25]). To further explore the similarities between zone-specific expression in human and mouse, we manually curated a list of zone-specific markers expressed in the mouse adrenal gland. *PTCH1*, *LAMB1*, *GNAQ*, and *ABCB1* were among the mouse marker genes exhibiting enrichment in their corresponding human zones (Fig. 2C), while other genes did not have spatially restricted expression or were either not expressed or did not have a human homolog (Supplementary Table S1 [25]). We were particularly interested in *RSPO3* and *WNT4*, which are members of the WNT signaling pathway and have been shown to regulate the balance between self-renewal and differentiation in the mouse adrenal gland capsule and zG, respectively [29]. Indeed, we found the spatial messenger RNA (mRNA) expression pattern of *RSPO3* (capsule) and *WNT4* (zG) in human and mouse adrenals was conserved (Fig. 2D). This observation prompted us to ask if the human adrenal sections recapitulated the known “WNT gradient” in which there is higher WNT activity in mouse zG relative to zF [29]. We used STdeconvolve to characterize the spatial organization of distinct cell types, or “topics,” and asked if any of them were enriched for WNT target genes using a previously published set of adrenal WNT target genes (*APCDD1*, *AXIN2*, *LEF1*, *LGR5*, and *NKD1*) [21, 24]. The spatial expression pattern of the topic enriched for WNT targets overlapped zG but not zF or zR (Fig. 2E). Although all 3 sections from the 31-year-old adrenal had a single topic enriched for WNT targets (slice B not shown), we did not find any enrichment in the 72-year-old adrenal section that was enriched for WNT targets, which could reflect the loss or depletion of the progenitor compartment. The observed transition between cell populations and the spatially restricted expression of conserved key adrenocortical regulators of self-renewal and differentiation suggest that the centripetal differentiation model determined in mice also underlies normal human adrenal zonation.

### The Transcriptome of Aldosterone-producing Micronodules

**Figure 3.**
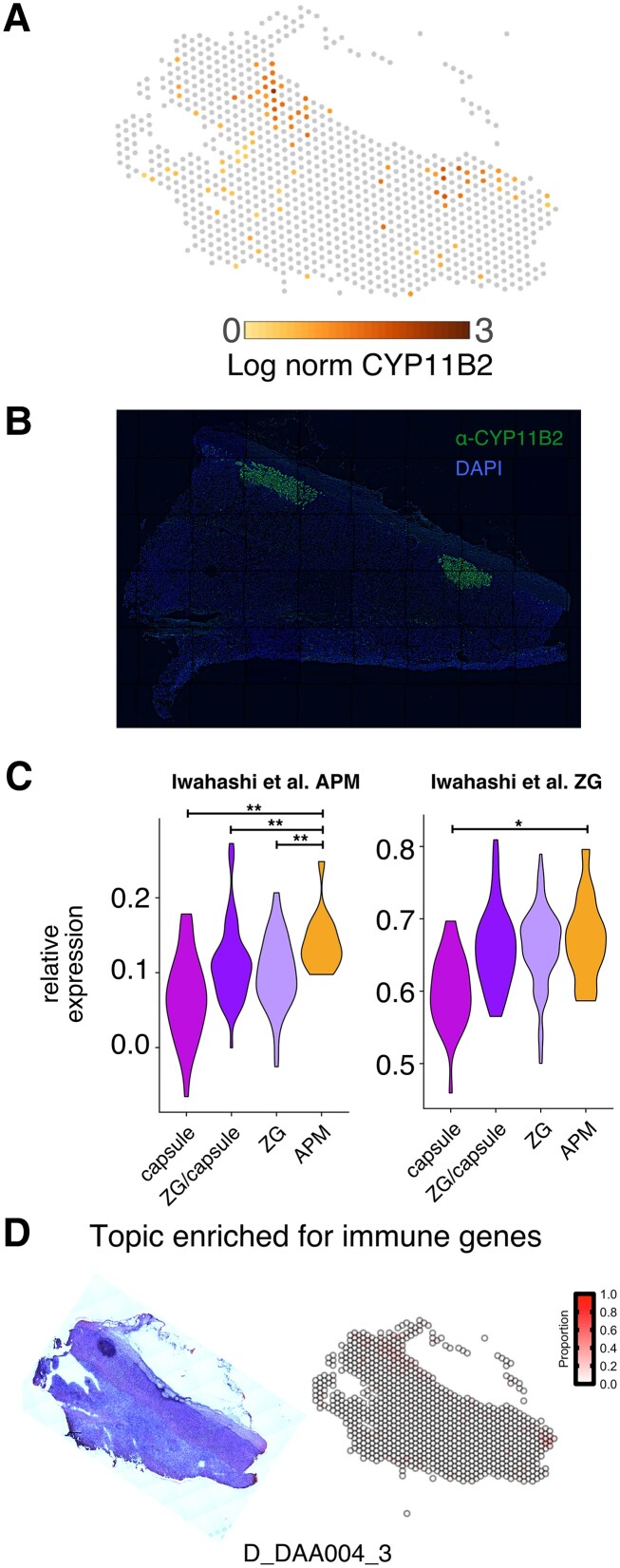
APM transcriptomic signature. A, *CYP11B2* messenger RNA expression for adrenal section corresponding to 72-year-old woman. B, CYP11B2 protein staining (green) and DAPI (blue) for adjacent adrenal section corresponding to 72-year-old woman. C, Violin plot of relative expression levels for genes associated with either APM (left) or zG (right) from Iwahashi et al. We performed pairwise tests between APM and all other populations but show only statistically significant relationships (*P < .05; **P < .01 Wilcoxon test). D, Hematoxylin-eosin stain (left) and heat map of the topic associated with immune markers (right).

APMs are an important example of spatially restricted cell heterogeneity in the adrenal cortex. APMs are defined by clustered high protein expression of the CYP11B2 enzyme (aldosterone synthase). In our samples, the *CYP11B2* mRNA (not shown) and protein expression (Supplementary Fig. S3A [25]) in the 31-year-old donor exhibited a typical continuous pattern across most cells of the zG layer. However, *CYP11B2* mRNA expression was discontinuous and localized to regional densities in the adrenal gland section from the 72-year-old donor (Fig. 3A). CYP11B2 protein by immunofluorescence on an adjacent section similarly revealed 2 positive staining regions consistent with multiple APMs (Fig. 3B). Interestingly, the spots containing APMs were most similar in gene expression to capsule/zG cell populations (Fig. 1A UMAP). Expression signatures of APMs have been suggested in previous studies either through analysis by single nuclei RNA-seq or laser capture microdissection. Iwahashi et al [28] defined a set of APM genes identified using single-nuclei RNA-seq that were significantly higher in our APM cell populations relative to other cortical cell populations (Fig. 3C, left). We did not observe a similar significant enrichment using gene sets identified using laser capture followed by microarray [30] (Supplementary Fig. S3B [25]). Moreover, the zG genes defined by Iwahashi et al [28] had similar expression levels in our APM and zG populations and lower levels in capsule (Fig. 3C, right), further supporting the specificity of the APM signature enrichment. We observed that *STAR* expression was higher in our APM cells compared with other cortex cells, indicating they have ample machinery of the rate-limiting step enzyme to produce excess aldosterone (Supplementary Fig. S3C [25]), consistent with the clinical picture in PA. Interestingly, each of the steroidogenic zone cell populations had a subpopulation with higher *STAR* expression, which may reflect further heterogeneity with respect to zone-specific hormone production. We characterized the transcriptome of APMs in a 72-year-old human adrenal tissue section that appears to have higher steroidogenic potential (ie, high *STAR* expression) and is enriched for genes identified in prior studies of APM-expression signatures.

We were also interested in examining other age-dependent differences in adrenal gene expression beyond the WNT pathway and APMs. Therefore, we compared spots annotated as zG between the 31- and 72-year-old donor adrenals to identify differentially expressed genes. We found 36 genes upregulated in the zG of the 72-year-old. Many of these genes were involved in lipid and steroid hormone metabolism, including *SCD*, *FADS2*, *HSD3B2*, *CYP11A1*, *CYP17A1*, *CYP11B1*, and *STAR*. Among the 101 downregulated genes were those involved in antigen processing and presentation (*HLA-A*, *HLA-B*, *HLA-E*, and *CD-74*), and the RNA-binding protein *ZFP36L2*, which is induced by angiotensin II and represses aldosterone production in H295R cells [31]. These results suggest that the adrenal exhibits age-dependent changes in immune cell recruitment and function, which is consistent with recent studies in mice [32]. Since we did not identify spots associated with immune cells, we again used the topics generated using STdeconvolve and searched for those overlapping immune markers (*CD33*, *MPO*, *FUT4*, *ELANE*, *CXCR2*, *CD14*, *S100A9*, and *S100A8*) from the human tissue, initially identified in mouse adrenals by single-cell RNA-seq [33], similar to our prior approach to assess WNT target genes (Fig. 2E). We found a topic enriched for immune markers in the 72-year-old adrenal but did not identify a topic in the sections from the younger adrenal gland even on increasing the number of topics to 50. Interestingly, the topic containing the immune marker genes was expressed adjacent to the APMs (Fig. 3D). These data support the existence of spatially defined, age-dependent differences in the expression of steroidogenic genes and presence of immune cells in human adrenal glands.

## Discussion

Mouse models are powerful tools to understand human development and disease. However, species-specific differences and the lack of human disease models are major barriers, especially for adrenal cortical cell differentiation, which still requires the development of bona fide human stem cell differentiation models [3, 5]. Thus, studying human tissue is an essential resource to understand adrenal cell differentiation and the dysregulation of that process leading to disease. Here we applied spatially resolved transcriptomics to normal adrenal tissue sections from a 31-year-old woman and a 72-year-old woman. We found that WNT pathway members required for proper cell differentiation, *RSPO3* and *WNT4*, have precisely the same spatially restricted expression in capsule and zG cells, respectively [26, 34]. Another interesting spatially restricted marker is *SRFP2*, secreted frizzled-related protein, which is a soluble modulator of WNT signaling expressed in the same cell populations as *WNT4*. SRFP2 is downregulated in APA, and loss of SRFP2 promotes aldosterone production through inhibition of WNT signaling in mice [35]. In spite of the fact that the some of the mouse adrenal cortex markers were not conserved in humans and that the mouse adrenal lacks a functional zR [30], our results indicate that the basic mechanisms and pathways driving the centripetal differentiation model is conserved in humans and mice, including the WNT activity gradient.

We identified multiple APMs in the zG region of the adrenal from the 72-year-old woman. When comparing 2 previously defined APM gene signatures, only one of them was enriched in our APM cell population [28, 36]. The lack of concordance between all signatures could be due to technical limitations or could indicate substantial heterogeneity existing between APMs from different individuals. Increasing the sample size, including sections from men, and continuing to span wide age ranges will be crucial to better understand APM heterogeneity and determine how it relates to PA and disease progression. The spot size of the Visium platform used in this study is 55 µm in diameter, which, therefore, includes contributions from multiple cells. We believe the lack of resolution made it difficult to identify high-confidence APM-specific genes and annotate individual spots as immune cell populations. However, we were able to use STdeconvolve to assess the positioning of immune cells, which were adjacent to the APM in the 72-year-old adrenal and completely absent in the 31-year-old adrenal sections. This suggests increased cortical immune cell infiltration with age, consistent with other studies [29, 37, 38]. However, the precise identity of the immune cells and whether their infiltration pattern is sexually dimorphic as seen in mice [29] remains to be determined in humans, further highlighting the need to expand sample demographics. This brings into focus the need to understand the dependencies between immune cells and age-dependent cortical changes and if immune suppression may mitigate this “inflammaging” phenotype [32].

In summary, a deeper understanding of normal adrenal function will help identify changes underlying both the APA and neoplastic processes. Recent multiomic technological developments combining spatially resolved DNA mutations [33] with RNA expression will be powerful for classifying and understanding adrenal pathologies. Nevertheless, this study provides evidence for age-dependent changes in human adrenal gene expression and spatial integrity of cortical zones that warrants future investigation.

## Data Availability

The spatial transcriptomics data have been deposited in the NCBI Gene Expression Omnibus (GEO) database and are publicly accessible through GEO accession number GSE244084. A browsable internet resource of the adrenal ST data, including H&E histology, clusters, and gene expression for all samples, is available at https://raysinensis.shinyapps.io/spatialshiny_adr/. Original data generated and analyzed during this study are included in this published article or in the data repositories listed in “References.”

## Abbreviations

APA: aldosterone-producing adenoma
APM: aldosterone-producing micronodule
H&E: hematoxylin-eosin
mRNA: messenger RNA
OCT: optical coherence tomography
PA: primary aldosteronism
UMAP: uniform manifold approximation and projection
zF: zona fasciculata
zG: zona glomerulosa
zR: zona reticularis
